# Empathy-related individual differences in brain responses to robot and human pain

**DOI:** 10.3389/fnhum.2026.1805177

**Published:** 2026-06-03

**Authors:** Jenna H. Chin, Kerstin S. Haring, Daniel Pittman, Pilyoung Kim

**Affiliations:** 1 Department of Psychology, University of Denver, Denver, CO, United States; 2 Department of Computer Science, University of Denver, Denver, CO, United States; 3 Department of Psychology, Ewha Womans University, Seoul, Republic of Korea

**Keywords:** empathy, fNIRS, human–robot interaction, prefrontal cortex, social robotics

## Abstract

**Introduction:**

Robots are increasingly implemented across diverse social contexts. Empathy is one construct that has gained focus in human-robot interaction (HRI) research. While previous studies have demonstrated neural activation for explicit depictions of robot pain, this work has primarily used a limited range of human and robot stimuli, and it is less clear how humans perceive a broader range of faces.

**Methods:**

The current study examined the neural correlates of human empathy for robot pain using functional Near-Infrared Spectroscopy (fNIRS) and a diverse range of human and robot face stimuli. In Study 1, we conducted an online survey with *N* = 63 adults to identify and validate photo stimuli of subtle depictions of robot pain. In Study 2, *N* = 39 adults completed an fNIRS task in which they viewed and rated human and robot photos in a painful touch and neutral conditions.

**Results:**

In Study 1, results found that the painful touch condition stimuli successfully elicited the intended emotion. In Study 2, there were no significant differences in brain responses to human pain in comparison to robot pain, though results demonstrated behavioral sensitivity to both the touch and face conditions. Neuroimaging analyses also suggest that individual differences in self-oriented empathy is linked to neural activation for human and robot pain.

**Discussion:**

The current work contributes our understanding of the role of individual differences in self-rated empathy in shaping neural responses to robot pain and highlights the importance of tailoring HRI to the emotional and empathetic responses of users. We did not find evidence for significant differences in neural responses to human pain stimuli in comparison to robot pain stimuli, despite significant differences in behavioral pain ratings. This suggests a discrepancy between brain and behavior necessitating further inquiry.

## 1 Introduction

In the past several decades, there has been an increased prevalence and implementation of social robots across a wide range of settings including healthcare, customer service, education, and even domestic use (Johal, 2020; Henschel et al., 2021; Ragno et al., 2023). In these contexts, robots have been used to interface with customers, provide social support and companionship, and teach or assist students with schoolwork (Šabanović and Chang, 2016; Pandey and Gelin, 2018; Foster and Petrick, 2020). These social robots have the ability to express emotions, gestures, and communicate with human users (Hegel et al., 2009). Importantly, the efficacy of these machines depends on their abilities to perform these social behaviors and actions in ways that are easily recognized by humans. The interdisciplinary field of human–robot interaction (HRI) has utilized perspectives from computer science, psychology, and neuroscience to understand human mind perception for robots in order to improve their interactive performance and capabilities. In particular, there is a growing interest in understanding the psychological processes and neural mechanisms involved in human perceptions of and interactions with robots.

Empathy has emerged as a central construct in the field of HRI, offering valuable insights into how humans perceive and engage with robots. Empathy is a complex construct with definitions ranging across disciplines from philosophy to neuroscience. In this present paper, empathy refers to “the capacity to understand and enter into another person’s feelings and emotions or to experience something from the other person’s point of view” (Colman, 2015). Extant literature on empathy has highlighted ways in which increased empathy is socially advantageous. For instance, previous research has shown that empathy increases social closeness and affiliative bonds (Morelli et al., 2015). Feelings of empathy can also motivate prosocial behaviors such as altruism (Bethlehem et al., 2017). Given these social implications, understanding the role of empathy is essential to enhancing interactions between humans and robots.

Prior HRI research has demonstrated that humans have the capacity for empathy toward robots, with certain features being particularly important in increasing empathetic feelings and behaviors. One primary contributor is anthropomorphism, the human-likeness of the robot (Fink, 2012). Foundational work on human mental models for robots have explored how people perceive robots with mechanical appearances in comparison to robots with anthropomorphic designs (Kiesler and Goetz, 2002). Although anthropomorphism is not limited to behavioral or visual similarities with humans and instead entails attributing any uniquely human characteristics (e.g., consciousness) to nonhuman agents (Waytz et al., 2010), previous design work has focused on heads and faces. Early work on humanoid robot design sought to identify the specific features of robot faces related to perceptions of human likeness (DiSalvo et al., 2002). Indeed, HRI studies have found that faces are particularly effective in eliciting empathy. For instance, individuals have reported higher feelings of empathy towards humanoid robots that have human-like features such as faces (Riek et al., 2009; Phillips et al., 2018) and emotional expressions or gestures (Gonsior et al., 2012). In line with this prior research, the current work utilized photo stimuli of robot faces to investigate the neural underpinnings human empathy for robot pain.

While behavioral research has demonstrated that humans are able to act empathically towards robots, less is understood about the neural underpinnings of these thoughts and behaviors. The current work sought to elucidate brain mechanisms of human empathy for robot face stimuli.

Prior neuroimaging studies on empathy among humans have demonstrated that several neural circuits comprised of subcortical and cortical regions are involved in empathy processes. The prefrontal cortex (PFC) is one specific cortical region that has been identified to play a central role in the cognitive and affective processes that underlie empathy (see reviews Walter, 2012; Engen and Singer, 2013; Marsh, 2018). In particular, previous neuroimaging work has demonstrated that PFC subregions such as the medial prefrontal cortex are involved in perspective-taking and mentalizing processes (Zaki et al., 2009). The dorsolateral prefrontal cortex has also been identified as playing a role in emotion regulation and modulating reactions to pain, including personal distress (Rêgo et al., 2015). One meta-analysis identified the dorsomedial prefrontal cortex as one member of the brain’s empathy for pain network (Lamm et al., 2011). The current study extended upon this foundational work on the neural circuitry of human empathy by investigating prefrontal cortex activation in the context of human empathy for robot pain.

Multimodal neuroimaging techniques have been used to investigate neural activation patterns of empathy for robots, typically employing visual paradigms designed to elicit empathy for pain. One early study using functional magnetic resonance imaging (fMRI) examined brain activation in response to video scenes of human and robots in friendly and torturing interaction, as well as a control condition featuring an inanimate object finding differences in neural activation in response to viewing human-human violent interactions compared to those of human-robot (von Rosenthal-Der Pütten et al., 2014). Research utilizing electroencephalography (EEG) has examined neural signatures of empathy, finding a shared neural signature of empathy for pain, regardless of agent type (Suzuki et al., 2015). EEG has also been used to study functional brain connectivity underlying human perceptions of robot pain (Chang et al., 2021). A more recent study used both fMRI and EEG to investigate neural responses for human and humanoid robot faces of pain and neutral expressions (Wu et al., 2024). Together, these studies provide initial insights into the neural correlates of human empathy for robot pain using visual task paradigms.

Despite this growing literature, it remains unclear how humans perceive pain across a broader range of human and robot faces, as previous work has used more limited stimuli. Notably, several previous studies did not use human or robot face stimuli. For instance, Suzuki et al. (2015) and Chang et al. (2021) presented first-person views of human and mechanical robot hands, while von Rosenthal-Der Pütten et al. (2014) used a robot resembling a baby dinosaur. Given that modern robots are designed to increasingly resemble humans, utilizing facial stimuli is particularly relevant to current and future design practices. Even more, increased anthropomorphizing of robots using face stimuli may be associated with increased empathic responses to these machines, not captured in previous work. Although the paradigm in Wu et al. (2024) did include human and humanoid robot faces, the human stimuli were limited to one racial background and one humanoid robot. As a result, neural responses to human and robot pain remain less understood across a more diverse range of human and robot stimuli that one can encounter in daily life. To address this gap, the current study utilizes a wide range of human faces varying in gender and race/ethnicity and multiple robot faces. This stimulus diversity enables a more comprehensive assessment of neural responses to pain cues across agents.

Importantly, individual differences may also play a significant role in shaping empathy for robot pain. Prior research in psychology and neuroscience has examined how individual differences in empathy contribute to neural variations, including differences in PFC function and structure. For instance, functional neuroimaging work has demonstrated that higher perspective taking, measured by the Interpersonal Reactivity Index (IRI; Davis, 1983), is associated with increased PFC activation during an emotion attribution task (Haas et al., 2015). One study of brain structure found a positive association between IRI fantasy scale scores and gray matter volumes in the right dorsolateral PFC (Banissy et al., 2012). A positive association has also been found between cognitive empathy and dorsomedial PFC gray matter volumes (Eres et al., 2015) as well as perspective taking and cortical thickness in several regions, including frontal ventrolateral regions (Uribe et al., 2019). Despite this prior work, there is still limited work examining the interplay of individual-level factors and the neural mechanisms of human empathy for robot pain. In the current study, we examine how differences in self-reported empathy may underlie brain responses to robot pain.

The present work aimed to investigate the neural correlates for human empathy to robot pain using a wide range of human and robot faces and subtle depictions of pain. To do this, we conducted two studies. In Study 1, we administered an online stimuli validation survey to identify a wide range of robot and human face photo stimuli depicting subtle pain manipulations that elicited the desired experimental condition (i.e., pain). These photos were then used as the photo stimuli for the neuroimaging task paradigm administered in the subsequent study. Study 2 consisted of an in-person experiment using functional Near-Infrared Spectroscopy (fNIRS) neuroimaging. fNIRS is a neuroimaging technique that measures hemodynamic responses as a proxy to neural activation (Ferrari and Quaresima, 2012). fNIRS is commonly employed in HRI studies, including studies examining prefrontal cortex activation in response to social robots (Hou et al., 2022; Lei and Rau, 2023; Achanccaray et al., 2025), as well as in research on neural responses to observed pain and empathy (e.g., Xie et al., 2018; Lian et al., 2025). Though to our knowledge, this approach has not yet been used to examine the neural mechanisms for robot pain specifically.

## 2 Study 1: stimuli development and validation

**Figure 1:**
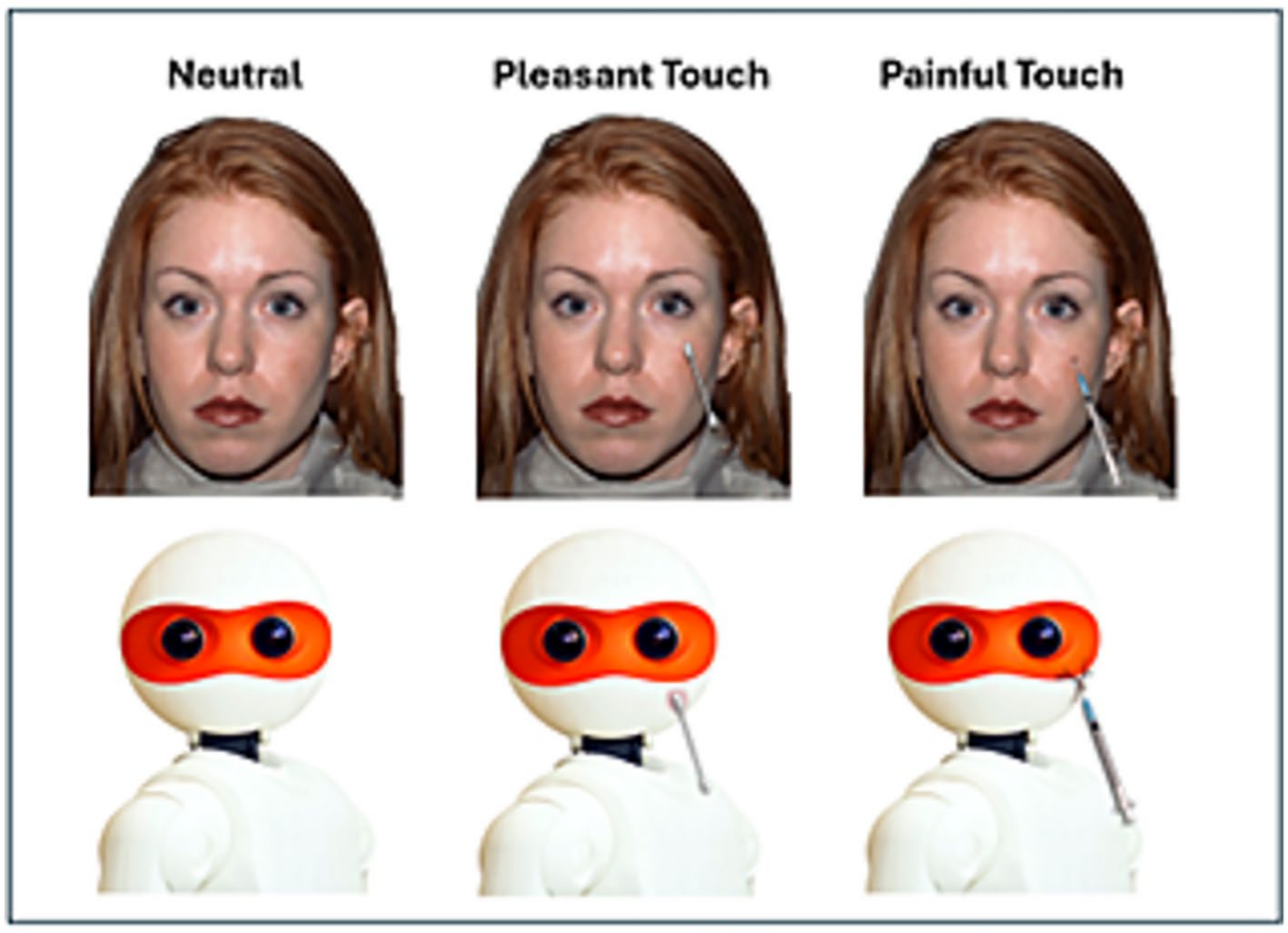
Example stimuli for each condition. Example stimuli for the human (top row) and robot (bottom row) face conditions across three touch conditions: Neutral (no touch), Pleasant Touch, and Painful Touch. The human face stimuli were obtained from the NimStim photoset (Tottenham et al., 2009), and the robot stimuli were obtained from The Anthropomorphic Robot Database.

In Study 1, participants completed an online survey where they viewed photos of human and robot faces with painful and pleasant touch manipulations. Stimuli were developed based on a study on cross-racial empathy (Sessa et al., 2014) which included a syringe needle and cotton swab. Although in the study conducted by Sessa et al. (2014) the cotton swab was used as a control condition, we opted to use it as a “pleasant touch” condition to examine responses across the full range of emotional valence, from aversive (“painful touch”) to pleasant (“pleasant touch). For both touch conditions, the photos were further manipulated where the object contacted the agent’s face. Specifically, a slight impression was added to human faces and crack was edited for the needle condition to show the needle penetrating the face, while a faint red glow was added for the cotton swab conditions (See Figure 1 for example stimuli). The goal of this study was to provide preliminary validation for the novel stimuli developed for the neuroimaging paradigm for the subsequent Study 2. We predicted that participants would provide a greater number of negative valence words in response to the painful touch condition stimuli and a greater number of positive valence words in response to the pleasant touch condition stimuli. We also predicted that the neutral stimuli would elicit neutral valence words.

### 2.1 Materials methods

#### 2.1.1 Participants

A total of 63 adults completed and were compensated for the online validation study. Participants were recruited from Amazon Mechanical Turk (MTurk), an online crowdsourcing platform commonly used in academic research (Aguinis et al., 2021). Of the 63 participants who completed the study, 4 participants did not complete the sociodemographic questions, resulting in missing demographic characteristics for these participants. Of the participants who provided demographic information, the sample was predominantly white (79.66%) and male (61.02%). The participants’ ages ranged from 26 to 72 years (M = 41.5, SD = 1.8) and had an average of 14.9 years of education (SD = 1.8).

#### 2.1.2 Study procedure

Participants completed the study via an online Qualtrics survey (Qualtrics, Provo, UT) administered via Amazon MTurk. Informed consent was obtained from all the participants. Following the completion of the survey, participants were compensated via the MTurk platform at the rate of $15.87 per hour, the minimum wage in Denver, Colorado at the time of data collection. The expected survey duration was 30 min. All online visit procedures were approved by the University of Denver Institutional Review Board, and all procedures were carried out in accordance with the relevant guidelines and regulations.

#### 2.1.3 Exploratory survey

The exploratory stimuli validation survey consisted of ratings and free-response word associations for 60 total images of human and robot faces, comprising of two agent types (human, robot) and three touch conditions (neutral/no touch, painful, pleasant). The human face stimuli were obtained from the NimStim photoset (Tottenham et al., 2009), a stimuli set widely used in human neuroimaging studies of emotion and affect. A total of 10 photos of adult faces were used in the present exploratory survey study. This set included five female and five male faces, and the racial/ethnic composition consisted of six white, three Black, and one Asian face. For a full listing of the stimuli used, please refer to Supplementary Table 1. The robot stimuli were obtained from The Anthropomorphic Robot Database (ABOT; version 04/09/2019; Phillips et al., 2018), a curated database of real-world robot images. For the current study, the research team selected a range of robots that were generally human-like (i.e., robots with identifiable facial features). Robots that might elicit strong emotional reactions (e.g., highly stylized or “cutesy” designs) were avoided. Robot images were also cropped to only include the head/face and upper body (i.e., “shoulders”) to match the human condition photos. Descriptive information and data-based provided human-likeness and facial feature scores for these stimuli can be found in Supplementary Table 2.

Images were digitally manipulated using Adobe Photoshop to include a needle or cotton swab object to indicate painful touch condition and pleasant touch condition, respectively. Photos without any manipulation were also presented as neutral condition stimuli. For the human face stimuli, the neutral face closed mouth expressions were used across all three conditions in order to match the neutral faces of the robot face photos. All photos were presented in color and at 360 by 360 pixels and 72 dpi.

**Figure 2:**
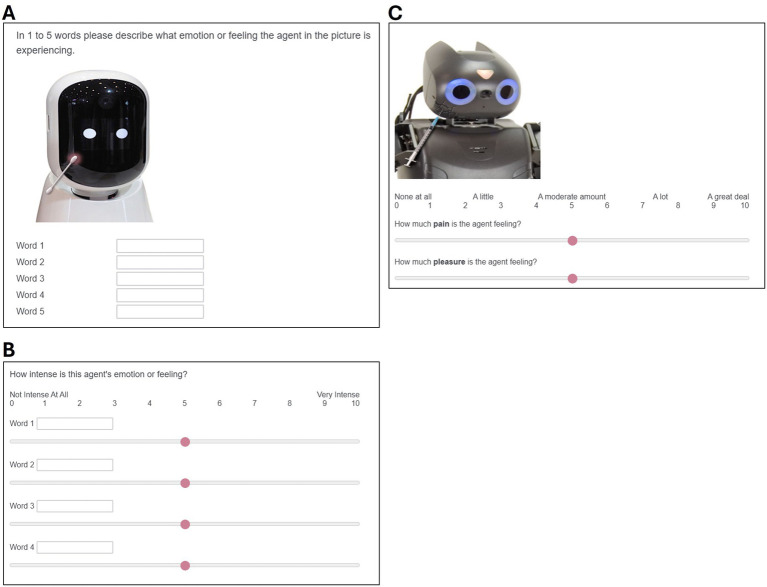
Example online survey block **(A)** Participants provided 1–5 words describing the emotion or feeling the agent appeared to be experiencing. **(B)** They then rated the intensity of each word on a 0–10 scale. **(C)** In the second portion, participants rated how much pain and pleasure the agent appeared to feel on a 0–10 scale. Sliders defaulted to the midpoint (“5”). Only responses from block **(A)** were used in the current analysis.

The online survey consisted of two parts. First, in the free-response portion, participants were asked to provide one to five individual words to describe the feeling or experience that the robot or human agent was feeling in the photo (Figure 2A). Participants were then asked to rate the intensity of each word they provided on a scale from 0 (“None at all”) to 10 (“A great deal”) (Figure 2B). In part two, participants were asked to rate how pain or pleasure each agent was feeling on a scale from 0 (None at all) to 10 (“A great deal”) (Figure 2C). For all numerical ratings, the slider default was set to 5 (“a moderate amount”). The current analysis is limited to the free association blocks. The survey concluded with questions on participant demographic characteristics.

#### 2.1.4 Data analysis

Free-response data were first inspected and cleaned prior to further analysis. Preprocessing steps included corrections for spelling, standardizing all words to lowercase, and removing invalid responses (e.g., “x” and “NA”). Following preprocessing, word frequency distributions were examined separately for each of the 6 conditions (i.e., robot painful touch, robot pleasant touch, robot neutral, human painful touch, human pleasant touch, and human neutral). The 10 most frequently provided words were identified for each condition. This full sample included a total of *N* = 9,384 words.

To assess whether response words aligned with the intended emotional manipulations, we examined their valence using the NRC Valence, Arousal, and Dominance (VAD) Lexicon Version 2.1 (Mohammad, 2018, 2025). The NRC VAD Lexicon comprises human ratings of valence, arousal, and dominance for over 55,000 English language terms. For the subsequent valence analysis, word responses were combined across agent type, for each condition (i.e., each condition included both human and robot face stimuli). The valence score was retrieved for each response word from the NRC VAD Lexicon, where valence scores for individual words range from −1 (negativeness/displeasure) to +1 (positiveness/pleasure), and scores closer to 0 indicate neutral valence. For example, the word “fear” has a valence score of −0.854, indicating a strong negative valence, whereas the word “happy” has a valence rating of 0.985, indicating strong positive valence.

To test whether the touch condition stimuli elicited the desired emotion from participants, a one-way Welch’s ANOVA was conducted to compare the effect of touch condition on the word valence scores. Words were excluded from the ANOVA analysis if not found in the lexicon. Across all three conditions, a total of *N* = 245 words were excluded from the analysis, resulting in a final sample of *N* = 9,139 words in the present analysis. Games-Howell post-hoc comparison tests were then performed to probe condition differences. Data preprocessing and statistical analysis were conducted in Python (version 3.10). Plots were generated in R (version 4.3).

### 2.2 Results

#### 2.2.1 Descriptive statistics and word frequencies

Prior to the valence score analysis, descriptive analysis was first conducted on the full sample of words. Total and unique word responses for each condition are provided in Table 1. Word frequencies were then examined for each experimental condition, and the top 10 words provided were identified. A summary of the top 10 more frequently provided words for each photo condition is shown in Tables 2, 3.

**Table 1:** Frequency of total and unique words provided for each touch condition.

Touch condition	Total	Unique
Robot pain	1,550	286
Robot pleasant	1,417	323
Robot neutral	2,035	392
Human pain	1,546	313
Human pleasant	1,428	322
Human neutral	1,408	392

**Table 2:** Frequency table of the top 10 words for each experimental condition for human agent stimuli.

Human					
Painful touch	Freq	Pleasant touch	Freq	Neutral no touch	Freq
Pain	167	Sad	92	Bored	83
Sad	110	Calm	56	Calm	82
Fear	60	Annoyed	52	Sad	77
Hurt	51	Fear	47	Neutral	66
Sadness	43	Bored	43	Annoyed	44
Annoyed	36	Sadness	37	Boredom	35
Anger	35	Neutral	34	Content	35
Calm	33	Anger	28	Anger	28
Neutral	32	Angry	26	Angry	28
Upset	32	Relaxed	25	Sadness	28

**Table 3:** Frequency table of the top 10 words for each experimental condition for robot agent stimuli.

Robot					
Painful touch	Freq	Pleasant touch	Freq	Neutral no touch	Freq
Pain	179	Fear	60	Neutral	87
Fear	101	Sad	57	Sad	63
Hurt	82	Neutral	45	Alert	63
Sad	74	Surprise	34	Calm	60
Sadness	40	Sadness	32	Happy	53
Anger	39	Pain	29	Fear	52
Broken	35	Happy	28	Interest	41
Surprise	34	Angry	27	Curious	35
Angry	32	Interest	27	Surprise	34
Upset	31	Calm	27	Content	32

#### 2.2.2 Word valence by touch condition

**Figure 3:**
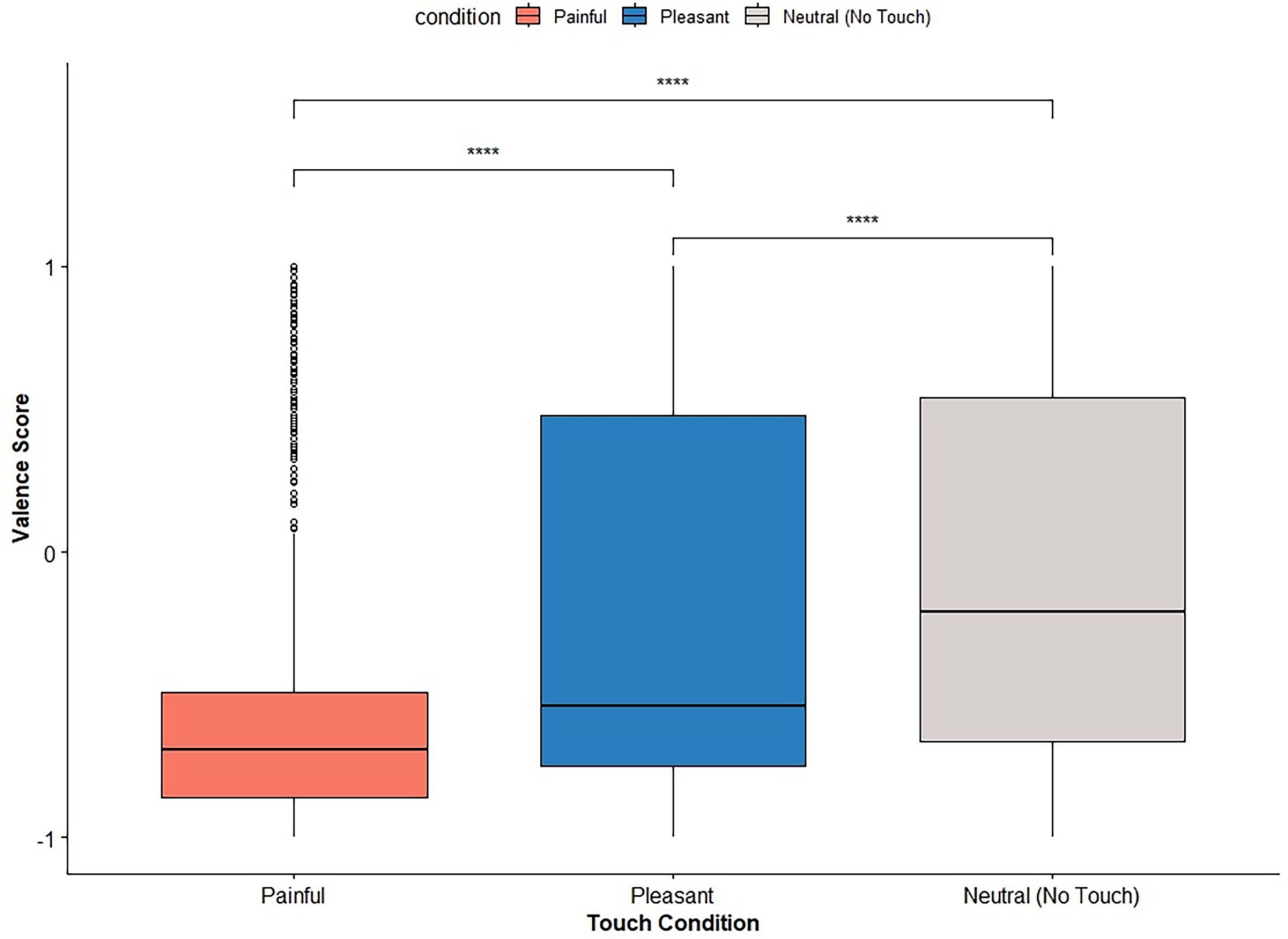
Box plots showing significant one-way ANOVA results valence scores of word responses in painful touch, pleasant touch, and neutral (no touch) conditions. Outlier values are represented by circles. ***p < 0.001.

Using a one-way Welch’s ANOVA to test whether the emotional valence of participant-provided words differed by stimuli touch condition (pain, neutral, pleasant), we found a significant effect of condition on valence scores *F*(2, 5,928.13) = 539.31, *p* < 0.001 (Figure 3). Follow up Games-Howell *post hoc* comparisons revealed significant differences between all pairwise tests. In line with our expectations, valence scores for words in the pleasant touch condition were significantly more positive than the painful touch condition (*p* < 0.001). Further, words provided for the neutral touch condition had significantly higher valence scores than painful touch condition words (*p* < 0.001). Unexpectedly, words provided in the pleasant touch condition had significantly greater negative valence scores than words in the neutral condition (*p* < 0.001).

### 2.3 Study 1 discussion

In Study 1, we conducted an online survey study of robot and human agent face stimuli with the aim of validating that the novel stimuli we developed elicited the desired emotions for each condition. The results indicated that while the painful touch stimuli successfully elicited the intended emotions across robot and human agents, the pleasant touch stimuli did not. Instead, responses to the pleasant touch stimuli predominantly resulted in negative valence and neutral emotional words. Based on these results, the pleasant touch condition stimuli were not used in the subsequent neuroimaging task, and we instead only tested painful touch and neutral conditions (no touch).

One potential explanation for the discrepancy between the pleasant touch photos and resulting responses is that the needle stimuli used in the painful touch condition were more visually clear and identifiable than the cotton swab used in the pleasant touch condition (Figure 1). This uncertainty in the pleasant touch manipulation may have led to confusion or discomfort, contributing to the unexpected negative emotional responses. Further, the free-word responses and valence approach provides an indirect measure of emotional perception. Future research should further explore how the clarity of stimuli and the type of touch influences emotional responses to robots across emotional conditions as well as explore alternative methods to more directly capture emotion perception.

## 3 Study 2: experimental fNIRS study

In Study 2, we examined differences in neural activation to human and robot pain employing the photo stimuli previously validated in Study 1. Extending upon extant functional neuroimaging literature on the neural correlates of empathy between humans, we focused on the prefrontal cortex (PFC), as this area includes brain regions implicated in theory of mind, cognitive empathy, and emotional empathy processes. To measure prefrontal cortex activation, we used fNIRS, a neuroimaging technique that estimates neural activation via changes in hemoglobin concentrations. fNIRS provides robust measurement of cortical hemodynamics, particularly in PFC regions. In comparison to EEG, fNIRS offers better spatial resolution. fNIRS is also more tolerant of motion than fMRI and allows for longer experimental sessions with lower discomfort or fatigue.

Study 2 consisted of two primary research questions. First, we tested whether there are differences in neural responses to human pain in comparison to robot pain. We hypothesized that there would be greater neural activation in brain regions associated with empathy and theory of mind in response to the human pain condition in comparison to the robot pain condition. Second, we examined how individual differences in self-report empathy may explain neural responses to human and robot pain. We hypothesized that greater self-reported empathy scores would be associated with greater prefrontal cortex neural activation in response to robot and human stimuli in the pain condition.

### 3.1 Materials and method

#### 3.1.1 Participants

A total of 40 participants were recruited from the University of Denver through an undergraduate psychology participant pool and flyers posted on campus. Inclusion criteria included: 18-years-old and older and English as a primary language. Exclusion criteria were a history of neurological disorder.

Overall, a total of 39 undergraduate and graduate student participants were included in the analysis. One participant was excluded from the analysis due to missing trigger data for the fNIRS task. This sample size is consistent with prior fNIRS studies on human-robot interaction (Le et al., 2022; Yorgancigil et al., 2022; Liu et al., 2024). Participants predominantly identified as white (79.5%) and female (59%), and their ages ranged from 18 to 29 years (M = 20.6, SD = 2.4). Participants completed an average of 13.3 years of education (SD = 1.7). See Table 4 for full sample characteristics.

**Table 4:** Study 2 sample characteristics.

Characteristic	*N* (%)	Mean ± SD	Range
Age (years)		20.6 ± 2.4	18–29
Education (years)		13.3 ± 1.7	12–20
RaceAsianBlack or African AmericanWhite/CaucasianOther	4 (10.3%)		
	3 (7.7%)		
	31 (79.5%)		
	1 (2.6%)		
EthnicityHispanic or Latino	4 (10.3%)		
SexFemale	23 (59%)		

#### 3.1.2 Study procedures

Participants completed an in-person lab visit at the University of Denver campus. Informed consent was obtained from all the participants. Participants then completed surveys on an iPad via REDCap (Harris et al., 2019). Participants were then introduced to the fNIRS system and Human Robot Faces fNIRS Task. Participants first completed an abbreviated practice version of the task to gain familiarity with the task instructions and stimuli timing. Participants were then fitted with an fNIRS cap and completed the Human Robots Faces fNIRS Task. Following the fNIRS task, participants completed additional ratings of the stimuli. Participants received either course extra credit or $40 cash for the completion of the study. All in-person study procedures were approved by the University of Denver Institutional Review Board, and all procedures were carried out in accordance with the relevant guidelines and regulations.

#### 3.1.3 Behavioral measures

##### 3.1.3.1 Interpersonal reactivity index

To assess individual differences in empathy, we used the Interpersonal Reactivity Index (Davis, 1983), a self-report questionnaire consisting of 28 items answered on a 5-point Likert scale ranging from “Does not describe me well” to “Describes me very well.” The measure comprises four scales each consisting of 7 different items. Scores for this measure are calculated separately for each scale. Perspective taking concerns one’s tendency to adopt the psychological point of view of others (M = 19.6, SD = 4.3). The Fantasy subscale includes questions related to one’s ability to imagine themselves having the thoughts and feelings of fictious characters (M = 18.2, SD = 4.4). Empathic Concern assesses other-oriented feelings of sympathy and concern for others (M = 20.1, SD = 4.4). Personal Distress evaluates self-oriented feelings of personal anxiety and unease in tense interpersonal settings (M = 11.2, SD = 4.9).

##### 3.1.3.2 Sociodemographic questionnaire

Participants completed a Sociodemographic Questionnaire survey which asked questions related to their demographic information such as sex, gender, race and ethnicity, and years of education.

#### 3.1.4 Human robot faces task

**Figure 4:**
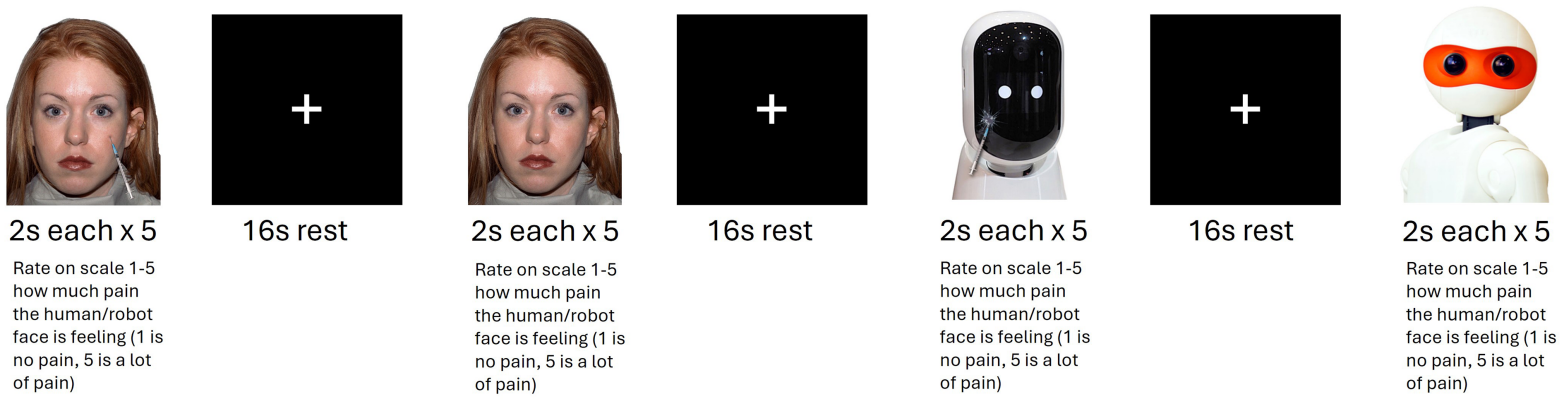
Human robot faces task paradigm. Example block design showing stimulus timing, rest periods, and rating prompt across task conditions. The human condition stimuli were obtained from the NimStim photoset (Tottenham et al., 2009), and the robot condition stimuli were obtained from The Anthropomorphic Robot Database (ABOT).

The Human Robot Faces Task (Figure 4) utilized the stimuli presented in exploratory survey conducted in Study 1. Like in the previously described survey study, a total of 10 human faces from the NimStim dataset and 10 robot faces from ABOT were used in the neuroimaging paradigm. The full list of stimuli used here can be found in Supplementary Tables 1, 2.

The task paradigm employed a block design of 5 same-group stimuli that were displayed for 2 s each, resulting in each stimulus block lasting 10 s in duration. Given that the pleasant touch condition did not elicit the expected emotion in the Study 1 stimuli validation survey (see Study 1 Results), the condition was not included in the final fNIRS paradigm. Thus, the task had four total conditions: human pain, robot pain, human neutral, and robot neutral. There were 12 blocks per condition and 16 s of rest (fixation cross) were displayed in between each block. Photo stimuli were randomized within each block. For each photo displayed, participants provided a rating from 1 to 5 indicating how much pain the face was feeling, where 1 indicated “no pain” and 5 indicated “a lot of pain.” This rating scale was modified from the Study 1 validation survey to better suit usability during the fNIRS task in the following ways: (1) the number of response options was reduced to accommodate the 2-s response window (2) the lower anchor for “no pain” was changed from 0 to 1 to align with the laptop’s numeric keyboard layout, in which 0 is positioned after 9 (rather than before 1).

#### 3.1.5 fNIRS acquisition and data analysis

**Figure 5:**
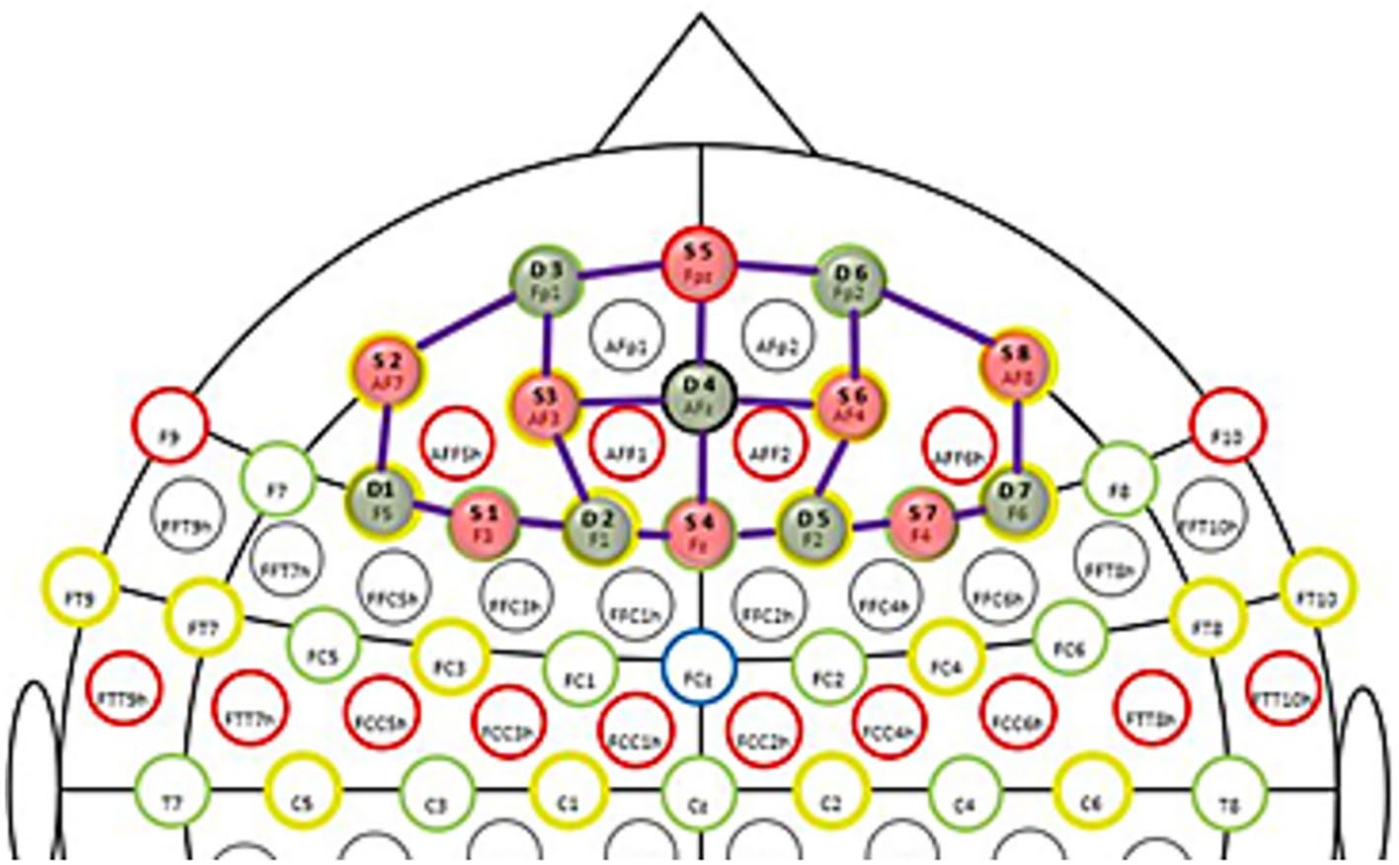
8 × 8 prefrontal cortex fNIRS montage sources are colored in red, and detectors are colored in green. Channels between source-detector pairs are indicated in purple.

fNIRS data was acquired using the NIRSSCOUT system (NIRx Medical Technologies, Berlin, Germany) with an 8×8 prefrontal montage consisting of 8 sources and 7 detectors with an approximate inter-optode distance of 3 cm. The montage includes 20 total channels (Figure 5). Prior to the start of the task, the NIRSCap was positioned approximately 1 inch above the participant’s brow line, and a signal calibration check was performed to ensure optimal signal for each optode. Two wavelengths (760 nm and 850 nm) were recorded to measure oxygenated and deoxygenated hemoglobin, respectively.

Data preprocessing and whole brain analysis was conducted in MATLAB 2019b (The MathWorks, Inc., Natik Massachusetts, USA) using NIRS Toolbox (Santosa et al., 2018) and custom scripts developed in collaboration with the NIRx team. Broadly, preprocessing steps included signal resampling, channel quality checking, hemoglobin conversation, and motion correction. Channel signal quality was assessed using the coefficient of variation (CV) threshold of 0.15 (Piper et al., 2014). Data were resampled from 10 Hz to 4 Hz, a standard preprocessing step to reduce high frequency noise (Santosa et al., 2018), and hemoglobin conversion was applied using a modified Beer Lambert Law (Huppert et al., 2006; Baker et al., 2014). Motion artifact correction was done using PCA filtering, which removes components of variance shared across channels that likely reflect global motion or physiological artifacts (Zhang et al., 2005; Santosa et al., 2018). First-level statistical analysis was conducted using a general linear model (GLM) with autoregressive, iteratively reweighted least squares (AR-IRLS) estimation, a robust method that accounts for temporally correlated noise and downweights statistical outliers to improve the accuracy of beta estimates (Barker et al., 2013; Santosa et al., 2018). Specifically, task regressors corresponding to each stimulus condition were modeled as 10-s stimulus blocks and convolved with a canonical hemodynamic response function (HRF) basis function to construct the design matrix. Beta values for each condition were then extracted for further analysis in IBM SPSS Statistics (Version 28). Extracted beta values were inspected for outliers. Outliers were identified as values greater than 3 standard deviations from the mean and were then Winsorized as 95%, an approach consistent with prior fNIRS studies (Kruppa et al., 2021; Papasideris et al., 2021). This approach was used in order to minimize the effect of extreme beta estimates while retaining all participants in the sample. Three values from two participants were replaced using this technique.

Second-level analysis was performed using a 2 × 2 Repeated Measures ANOVA to analyze the effect of agent face (human, robot) and condition (neutral, pain) on the neural activation of each channel. We examined both oxygenated (HbO) and deoxygenated (HbR) hemoglobin, where higher HbO values and lower HbR values reflect greater brain activation (Villringer et al., 1993). To test differences in neural activation to robot pain compared to human pain, no covariates were added to the ANOVA model. As a baseline estimate of power, a *post hoc* power analysis was conducted in G*Power and indicated that our sample was adequately powered (>0.8) to detect medium effects (*d* = 0.05) for a 2 × 2 repeated measures ANOVA without a covariate. To examine the role of individual differences in empathy on neural activation, separate models were tested in which each Interpersonal Reactivity Index scale score was included as a covariate. Corrections for multiple comparisons was performed using the Benjamini-Hochberg procedure (Benjamini and Hochberg, 1995) and applied separately to each family of tests (e.g., main effects and interactions) across all 20 channels, with an adjusted *p*-value of 0.0025. Post-hoc Pearsons correlations were used to decompose significant interactions. We used fNIRS Optodes’ Location Decider (fOLD) (Zimeo Morais et al., 2018) as well as corresponding Brodmann areas for further anatomical and regional interpretation of significant channels.

### 3.2 Results

#### 3.2.1 Participant characteristics and correlations

Correlations between main variables can be found in Table 5.

**Table 5:** Correlation table of main study variables.

Variable	1	2	3	4	5	6	7	8	9	10
1. Age	1									
2. Education	0.772**	1								
3. Human Neutral Rating	0.21	0.01	1							
4. Human Pain Rating	0.162	−0.017	0.570**	1						
5. Robot Neutral Rating	0.12	−0.026	0.736**	0.378*	1					
6. Robot Pain rating	0.338*	0.091	0.556**	0.481**	0.624**	1				
7. IRI Fantasy Scale	−0.221	0.022	0.021	0.026	0.032	0.079	1			
8. IRI Perspective Taking Scale	0.247	0.114	−0.056	−0.142	−0.12	0	−0.111	1		
9. IRI Empathic Concern Scale	0.143	0.332*	−0.008	−0.241	−0.066	−0.106	0.420**	0.137	1	
10. IRI Personal Distress Scale	−0.238	−0.199	0.207	−0.009	0.379*	−0.109	0.17	0.421**	−0.039	1

**Correlation is significant at the 0.01 level (2-tailed). *Correlation is significant at the 0.05 level (2-tailed).

#### 3.2.2 Human robot faces task pain ratings

**Figure 6:**
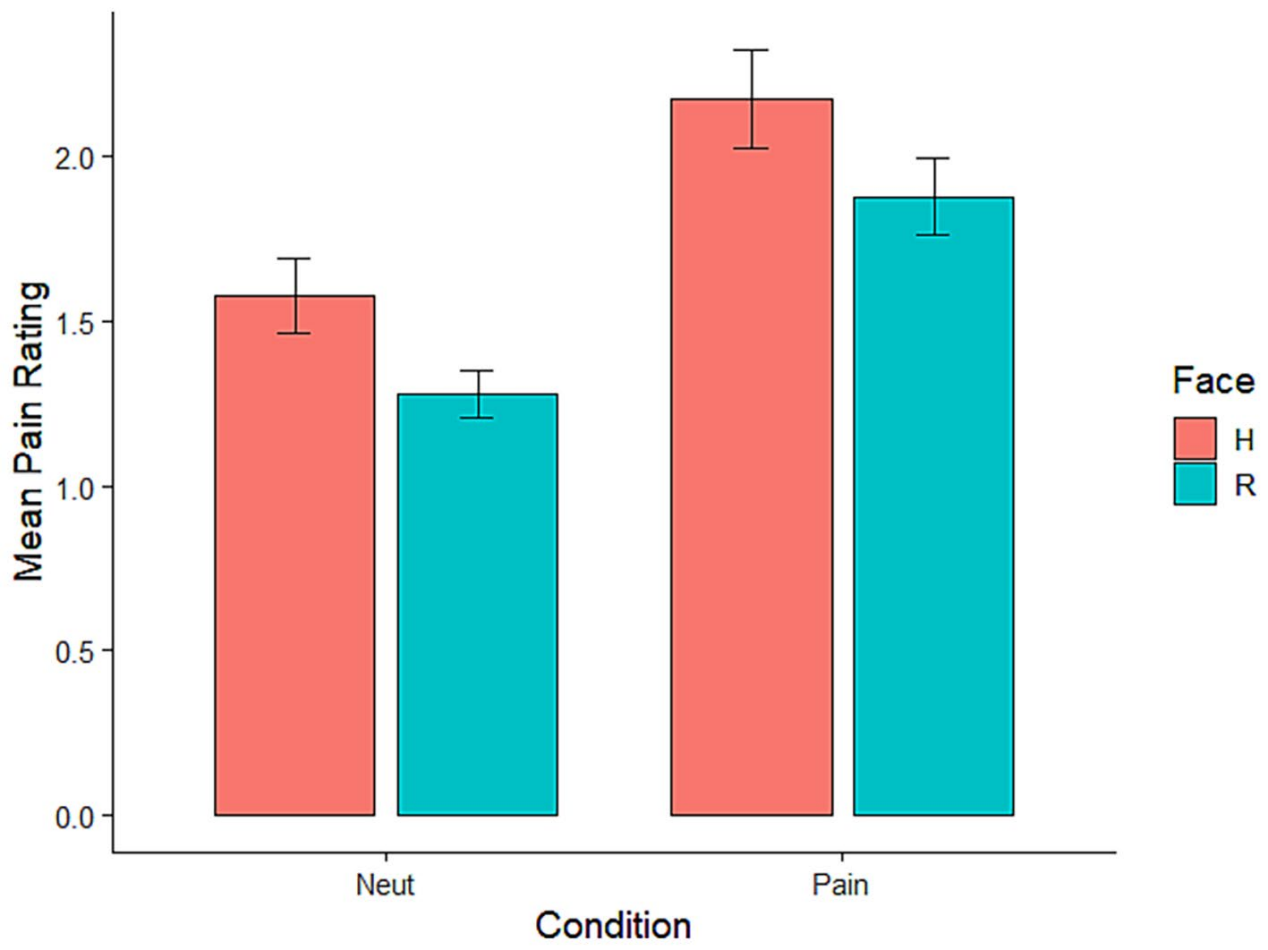
Effects of agent face and condition on pain ratings full factor bar chart displaying average pain ratings across agent face (human vs. robot) and condition (neutral vs. pain).

Using a 2×2 Repeated Measures ANOVA testing the effect of agent face (human, robot) and condition (neutral, pain) on pain rating, we found a significant main effect of condition such that participants rated pain stimuli as experiencing more pain than neutral faces *F*(1, 38) = 48.22, *p* < 0.001. Further, there was a significant difference for the main effect of face such that human faces were rated as experiencing more pain than robot faces *F*(1, 38) = 11.08, *p* = 0.002 (Figure 6). There were no significant interaction effects *F*(1, 38) = 0.00, *p* = 0.997.

#### 3.2.3 Differences in neural activations to human vs. robot pain

To test whether there exists differences in neural responses to human pain in comparison to robot pain, we conducted a 2 × 2 Repeated Measures ANOVA analyzing the effect of agent face (human, robot) and condition (neutral, pain) on the beta activation of each channel. There were no significant main effects of agent face or touch condition, nor any significant interaction effects for either HbO or HbR signals (all *p*s > 0.05). Additional details for this analysis can be found in the Supplementary Data.

#### 3.2.4 Individual differences in empathy and neural activations to human vs. robot pain

**Figure 7:**
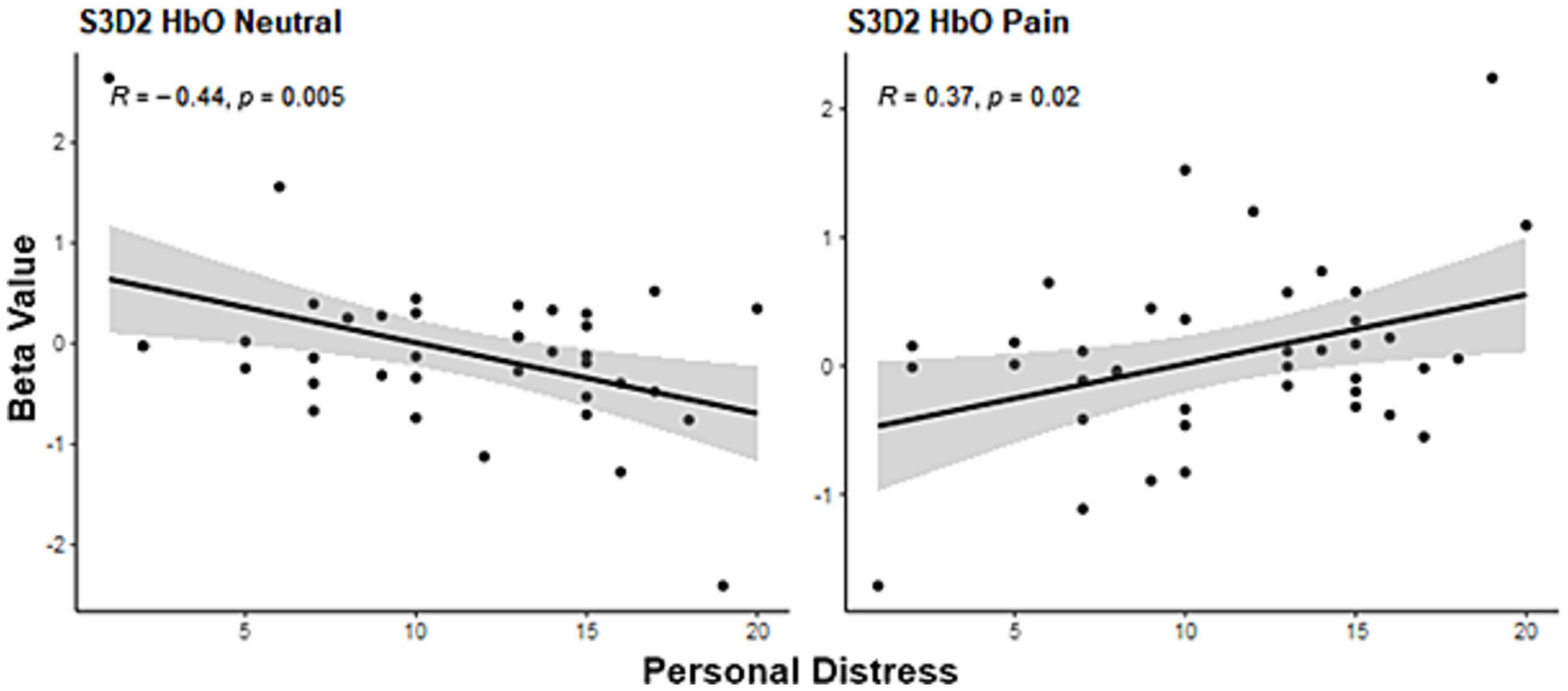
Association between personal distress and HbO beta values in S3 D2. Scatter plot of the relationship between IRI personal distress scale scores and S3D2 HbO beta values in response to the neutral (left) and painful touch (right) task conditions, across humans and robots.

**Figure 8:**
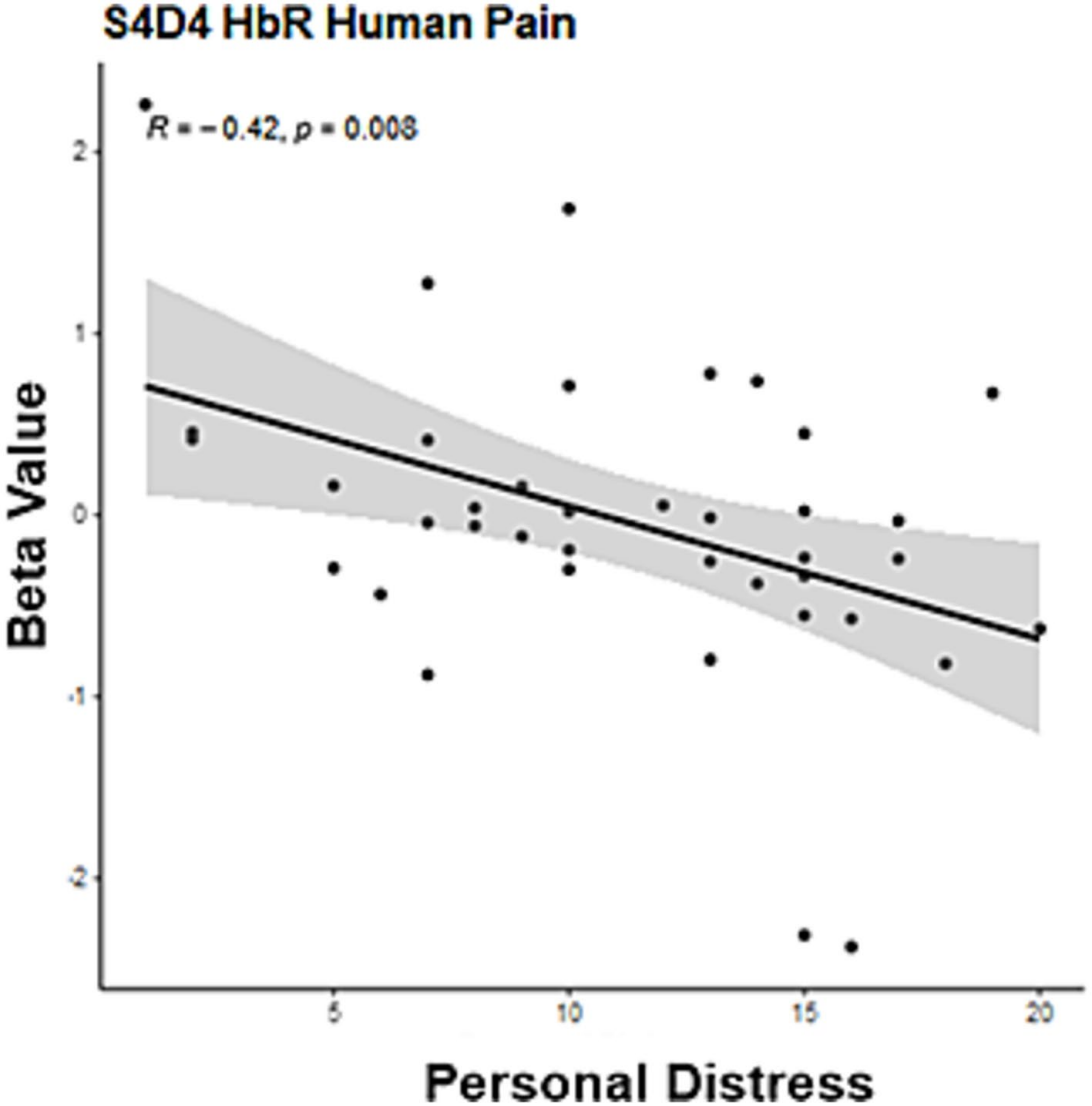
Association between personal distress and HbR beta values in S4 D4. Scatter plot of the relationship between IRI personal distress scale scores and S4D4 HbR beta values in response to the human painful touch task condition. Lower HbR values reflect greater brain activation.

To examine whether individual differences in self-reported empathy were related to neural responses to human and robot faces, we conducted four separate 2×2 Repeated Measures ANOVAs, covarying for each empathy scale. For the oxygenated hemoglobin response (HbO), there was a significant two-way interaction in channel S3D2, *F*(1, 37) = 12.78, *p* < 0.001, such that higher personal distress scores was associated with less activation to neutral faces and more activation to pain faces, across both human and robot faces (Figure 7). For the deoxygenated hemoglobin response (HbR) we found a significant three-way interaction in channel S4D4 *F*(1, 37) = 10.82, *p* = 0.002, such that higher personal distress scores was associated with lower HbR to human painful touch (Figure 8). These results survived the multiple comparison correction. There were no other significant effects for the other empathy scales (*ps* > 0.05).

### 3.3 Study 2 discussion

In Study 2, we examined the neural correlates of human empathy for robot pain in the PFC using fNIRS. Inconsistent with our hypothesis, we did not find a significant difference in neural responses to human painful touch pictures in comparison to robot painful touch pictures. Unexpectedly, results also indicated no significant differences in neural activation between neutral (no touch) and painful touch images regardless of agent face. Behaviorally, however, we found significant main effects for both touch condition and agent face, such that participants rated faces in the painful touch condition as experiencing more pain than those in the neutral condition and human faces as experiencing more pain robot faces. This behavioral finding demonstrates that the pain condition manipulation was successful in eliciting empathic responses to pain, though not at the neural level in the PFC. Our null finding contributes to the growing literature on the complex relationship between behavioral and neural empathy responses to pain, highlighting the need for further research to tease apart this discrepancy. For instance, previous fMRI work has demonstrated a lack of significant correlation between participant’s self-reported pain sensitivity and brain activation (Hoeppli et al., 2022), and prior EEG research has shown that that subject pain or empathy ratings were not related to neural activity (Camara et al., 2026). Further, this lack of significance difference in PFC neural activation may suggest the involvement other brain regions implicated in empathy and theory of mind processing, warranting further investigation.

We also did not find a significant difference in PFC responses between agent face across touch conditions. This lack of significant differences in neural responses is similar to previous work where there were no differences in neural activation patterns were found in response to videos of human-human interaction in comparison to human-robot interaction across interaction type (i.e., affectionate vs. violent; von Rosenthal-Der Pütten et al., 2014). As previously discussed, since the current study focused exclusively on the PFC, it remains possible that differences in activation to human and robot faces may be observed in other brain regions. For instance, prior work has reported differential activations in regions such as the temporoparietal junction in response to humanoid robot pain stimuli (Wu et al., 2024).

Moreover, the design of the task paradigm may have also contributed to the lack of significant findings. Specifically, the inclusion of a wide variety of robot and human face stimuli within each condition may have introduced increased variability between stimuli that concealed distinct patterns of neural activation. Because we intended to match the human and robot facial stimuli closely, differences in visual appearance between robot and human stimuli were not as apparent. In addition, the subtle visual manipulations of pain as indicated by the needle is less salient than previous work using more obvious depictions of pain, such as video clips.

We next examined whether individual differences in self-reported empathy may underlie neural responses in the PFC to robot pain. This work extends upon prior research on the role of individual differences in shaping interactions with robots (Syrdal et al., 2007). Overall, we found that individual differences in the personal distress scale of the Interpersonal Reactivity Index (IRI) (Davis, 1983), a measure of self-oriented feelings of distress and anxiety in response to the distress of others, may shape PFC responses to human and robot pain stimuli.

In channel S3D2, we found that higher personal distress is associated with lower activation to neutral faces and more activation to painful touch faces, across both the human and robot conditions. This channel includes the left dorsolateral prefrontal cortex (DLPFC; BA 46) as well as left portions of the dorsomedial prefrontal cortex (DMPFC; BA 9). Previous studies show that the DLPFC plays a role in modulating affective and empathic responses to pain (Wang et al., 2014; Balconi and Canavesio, 2016). Within this work, the DLPFC has been more specifically implicated in the suppression of pain, with one study finding greater activation of the DLPFC, in response to acute pain stimulation, when participants were directed to suppress pain (Freund et al., 2009). Our result may indicate that those with higher levels of personal distress employ greater effort to regulate or suppress their feelings of distress in response to visual pain stimuli. Additionally, this effect was observed across both human and robot faces, suggesting a shared neural mechanism in the DLPFC that supports the regulation of distress in response to pain. This is consistent with previous work demonstrating a shared neural mechanism for processing pain across humans and robots, evidenced by an overlapping neural mechanism for robot and human pain in the P3 component (Suzuki et al., 2015).

In channel S4 D4, we found that higher levels personal distress was associated with lower deoxygenated hemoglobin response during the human painful touch condition. While S3D2 includes a significant amount of the lateral PFC regions, S4D4 is located in the medial region including the DMPFC (BA 9) as well as portions of the frontopolar (BA 10) and frontal eye field (BA 8) regions. A decrease in HbR is traditionally thought to occur during brain activation (Villringer et al., 1993), with these results suggesting that individuals with higher personal distress related empathy show greater DMPFC recruitment in response to human pain stimuli. The DMPFC plays a central in theory of mind processes and is activated when individuals engage in perspective-taking, infer others’ mental states, and distinguish between self and others (D’Argembeau et al., 2007). In this specific brain regions, this effect was found to be specific to depictions of human pain, suggesting that theory of mind processes related to personal distress are particularly sensitive to processing human pain. Prior research has demonstrated “in-group” bias in theory of mind processing (Zhu et al., 2023), and research in evolutionary psychology provides biological rationale for a tendency to empathize with in-group members (De Dreu and Kret, 2016). Further, research has also shown the role of the DMPFC in emotion memory (Kensinger and Ford, 2021) with associations with empathy processing (Tani et al., 2014). Interactions between autobiographical memory and empathy may be more sensitive to human pain, with robot faces evoking these associations less strongly. However, as this association between S4D4 and personal distress was observed in HbR only, and not in HbO, the finding should be interpreted with caution. Future studies are needed to further investigate how oxygenated and deoxygenated hemodynamic responses are related to individual differences in empathy.

Lastly, several methodological considerations should be taken into account when interpreting the present neuroimaging findings. First, PCA filtering was applied during motion correction procedures; however, other preprocessing approaches commonly used in fNIRS research to further reduce motion-related and physiological noise, such as the use of short-separation channels during data acquisition, were not implemented in the current study. Second, although the study was adequately powered to detect medium-sized effects in the primary repeated-measures ANOVA model, the inclusion of multiple covariates, channel-wise analyses, and correction for multiple comparisons may have reduced sensitivity to detect smaller or more subtle effects. Finally, beta-value Winsorization was used to reduce the influence of extreme values while retaining participant data, though alternative outlier-handling approaches, including case-wise exclusion, are also commonly employed in the fNIRS literature.

## 4 General discussion

Understanding how individual differences in empathy may underlie human empathy for robot pain is important in improving the design and implementation of social robots. Our study aimed to build on previous research by examining neural activation in response to a broader range of human and robot facial expressions, addressing the limitations of prior work that primarily focused on a narrow set of stimuli depicting explicit robot pain. First, we conducted an exploratory survey study to identify and develop a wide range of human and robot face stimuli with subtle visual pain manipulation. Using these stimuli, we then conducted an fNIRS neuroimaging study to examine the neural correlates of human mind perception for robots. There were no significant differences in neural responses within the PFC to human vs. robot faces or painful touch vs. neutral conditions, though the paradigm elicited behavioral sensitivity to both agent type and touch condition. However, we found evidence that suggests individual differences in self-oriented empathy may underlie neural responses in dorsolateral prefrontal cortex (DLPFC) and dorsomedial prefrontal cortex (DMPFC) brain regions to human and robot pain.

The current study possessed several strengths. Most notably, the paradigm stimuli were a key strength, including their development, validation, and content. The task paradigm incorporated a broad range of face stimuli for both human and robot agents, which were further externally validated by an independent sample of raters. Additionally, our study emphasizes individual differences in empathy, allowing for the examination of how participants’ trait-level empathy modulated neural responses. This work also possessed methodological strengths. As a neuroimaging modality, fNIRS enables participants to perform tasks outside of an MRI scanner, similar to EEG studies, but with greater spatial resolution.

This study should also be interpreted within the context of its limitations. Firstly, although this study sought to employ a broad range of human and robot stimuli, only static faces with neutral expressions were shown. This was done to ensure consistency across the robot and human condition but resulted in the experience of pain being implied rather than explicitly shown. Additional work should be conducted utilizing even broader stimuli, including dynamic stimuli such as video and auditory stimuli, or even real robots. Second, our study also restricted our investigation of brain activation to the PFC. Although the PFC includes regions implicated in empathy processes, research has also highlighted the involvement of other brain regions, such as the anterior insula and anterior cingulate cortex, regions integral to the processing of affective states and emotional responses (Singer et al., 2004; Lamm et al., 2011). Third, participants were limited to undergraduate and graduate students primarily studying psychology and computer science who may have increased exposure to robotics. Additional work is needed to explore these associations in a broader sample to increase generalizability. Prior work has also found age-related differences in behaviors and feelings towards robots (Martínez-Miranda et al., 2018), thus it may be important to examine the role of empathy across age ranges. Lastly, although the Interpersonal Reactivity Index has demonstrated psychometric validity and reliability, it is a self-report measure of empathy which may be vulnerable to reporting bias. Additionally, separate models were run for each subscale rather than a single combined model, which may increase the risk of Type I error due to multiple testing.

By examining the neural mechanisms of human empathy, this work contributes to the broader goals of improving the design and implementation of social robots across diverse social settings. This study utilized a wide range of robot and human face stimuli, and future work should continue to use a wide range of stimuli, including real robots rather than only photos. Even more, questions remain regarding how understandings of individual differences can be effectively integrated into robots’ behavior and response systems to optimize interactions across various user groups. Future work in robot design and implementation could explore how robots can adapt in real-time to different levels of empathy in users.

The current work also underscores the need for tailoring robot interactions to the emotional and empathetic responses of users. Previous work has developed guidelines for robot designs intended for empathic interactions (Breazeal et al., 2016). Extending upon this, our findings suggest that individuals with higher levels of empathy may be more attuned to robots’ emotional states, an important consideration for the design of robots in caregiving, healthcare, and educational environments. Increased sensitivity may be beneficial such that these individuals are able to form strong bonds in caregiving settings or show higher engagement in education settings. However, high levels of personal distress in response to robot pain may result in maladaptive interactions with robots such as difficulties in regulating negative emotions. This is further supported by previous research on the ethical implications of emotional relationships with robots (Darling, 2016) as well as broader discussions of robot ethics related to rights and boundaries (Gunkel, 2018). These findings may have particular relevance for the design of more anthropomorphized robots, as greater human-like facial features could enhance empathic engagement while also raising important ethical considerations.

## 5 Conclusion

The current work provides a further understanding of the role of individual differences in empathy in shaping neural responses to robot pain. Using a novel set of stimuli validated by an independent sample of raters, our research suggests that individual differences in self-oriented empathy may underlie differential brain activation to human and robot pain. Though participant behavioral ratings differed significantly between face types and pain condition, we did not find evidence for significant differences in neural responses within the PFC to human pain stimuli in comparison to robot pain stimuli. Our results offer preliminary insight suggesting that individual differences in empathy may shape neural responses to human and robot pain. The findings from this work have the potential to inform the design and implementation of social robots.

## Data Availability

The datasets presented in this article are not readily available because they contain information that could compromise research participant consent and privacy. Due to the sensitivity of the human participant data and the potential for identification of de-identified participant, it would be important to make the data available to other researchers under a data-sharing agreement that provides for a commitment to data security approved by the corresponding author’s institution IRB. Requests to access the datasets should be directed to pilyoung.kim@du.edu.
