# Supplementary material for: Empathy-related individual differences in brain responses to robot and human pain
Source: Front Hum Neurosci. 2026 Jun 3;20:1805177. doi: 10.3389/fnhum.2026.1805177 (PMC13272435; doi:10.3389/fnhum.2026.1805177)
Supplement: Supplementary file 1 [file Data_Sheet_1.docx]

Supplementary Material

# Supplementary Data

We conducted separate 2×2 repeated measures ANOVAs for each of the 20 fNIRS channels in the montage, with factors Agent Face (Human, Robot) and Condition (Neutral, Pain). Both HbO and HbR signals were analyzed. Across all channels, there were no significant main effects of agent or condition, and no significant agent × condition interactions (all *ps* > .05).

# Supplementary Tables

## Supplementary Table 1 (NimStim Human Faces)

| **Gender** | **Race** |
| --- | --- |
| Female | White |
| Female | Black |
| Female | White |
| Female | Black |
| Female | Asian |
| Male | White |
| Male | White |
| Male | White |
| Male | White |
| Male | Black |

## Supplementary Table 2 (Anthropomorphic Robot Database)

| **ABOT ID** | **Name** | **Image** | **Human-Likeness Rating** | **Facial Features Score** |
| --- | --- | --- | --- | --- |
| 84 | hub | 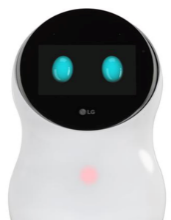 | 15.87 | .654 |
| 21 | darwin-op | 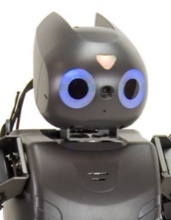 | 40.04 | .736 |
| 40 | Pr2 | 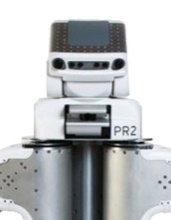 | 20 | .706 |
| 42 | mip | 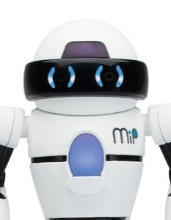 | 30.22 | .708 |
| 63 | Nimbro-op humanoid | 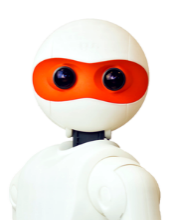 | 48.2 | .701 |
| 83 | Sawyer | 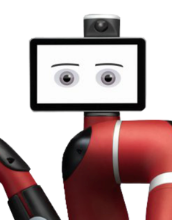 | 16.92 | .667 |
| 107 | aila | 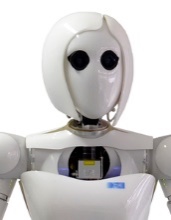 | 43.26 | .729 |
| 164 | aido | 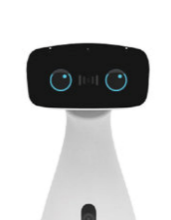 | 10.32 | .679 |
| 207 | otto | 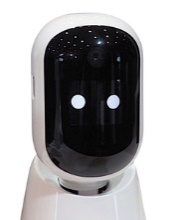 | 20.66 | .51 |
| 215 | Av1 | 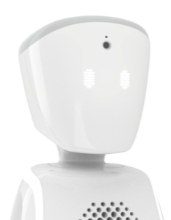 | 14.04 | .57 |
